# A Proving of Ustilago Maidis

**Published:** 1889-06

**Authors:** S. Swan

**Affiliations:** New York


### A PROVING OF USTILAGO MAIDIS.

S. SWAN, M. D., NEW YORK.

Gave Mrs. —— *Ustilago*$^{1m}$ (Fincke), four doses for aching distress and extreme soreness of os uteri. After twenty-four hours she was cured, but had the following symptoms, never before felt by her: Headache in temples. Pains at root of nose, extending in toward canthi, and up and out at each eyebrow. Pâin in back of neck. Great pains in bones all over body, and especially in calves, which are somewhat cramped. Pain in both shoulders, especially on raising arms. Stiffness in shoulder-joints on bringing down the arms on waking—the arms were extended over head when sleeping. Thirst for cold drinks. Felt chilly externally, but not internally. Frequent urination, with pain at meatus as the last drops were passing.

